# Supplementary material for: Evaluating the impact of Hazelwood mine fire event on students’ educational development with Bayesian interrupted time-series hierarchical meta-regression
Source: PLoS One. 2023 Mar 1;18(3):e0281655. doi: 10.1371/journal.pone.0281655 (PMC9977026; doi:10.1371/journal.pone.0281655)

# Supporting Information I - Tutorial for Bayesian interrupted time series hierarchical meta-regression

Import library packages

```
library(pacman)
p_load(c("tidyverse",  "rstan", "bayesplot","kableExtra"), character.only = TRUE)
```

### Import data

The data as well as the analysis code used in this tutorial can be directly downloaded from Github repository: https://github.com/CarolineXGao/NAPLAN_impact.

```
# use file path of saved NAPLAN data
naplan <- read.csv(here::here("Data","naplan_fake_data.csv"))
```

The variables in the data set are:

| Variable name | Details |
|---|---|
| ID | School ID |
| Cohort_ID | Cohort ID, which reflects the same cohort of students during NAPLAN assessment (e.g. grade 3 2003, grade 5 2005, grade 7 2007, grade 9 2009) |
| Year | Year of NAPLAN assessment |
| Grade | Grade of students who completed NAPLAN assessment |
| Domain | Domain of NAPLAN assessment, this domain is grammar and punctuation |
| ICSEA | Index of Community Socio-Educational Advantage per school and year |
| N_student | Number of students enroled in the school in the year of NAPLAN test |
| P_girls | Proportion of female students in the school and year of NAPLAN test |
| Government | Whether the school is a government school |
| Exposure_group | Location of school as a proxy for coalmine fire exposure: Morwell schools - High exposure, non-Morwell Latrobe Valley schools - Moderate exposure, and Wellington Schools - No/low exposure |
| ScoreDif | Mean NAPLAN score of the school - Victorian regional schools mean score for the matching year and grade level (mean NAPLAN score difference) |
| SE | Standard error of mean NAPLAN score for the school, year and grade level |

## Modelling

### Prepare data for modelling

A number of variables need to be changed for Bayesian modeling with Stan. Categorical variables, including grade (Grade) and exposure group (Exposure_group), need to be re-coded as dummy variables. Binary variables should be 0 or 1 (Government vs non-Government). In order to intemperate intercept of the model here we also center the numeric variables at the mean value and year at the start of the cohort (2008). Finally, interaction effects need to be created prior to the modelling. Grade 7 and 9 were combined due to relatively smaller numbers. Also the estimated effect size were also very similar when included separately.

```
naplan <- naplan %>%
  mutate(
         # years after and during coalmine fire
         Fire = ifelse(Year >= 2014, 1, 0),
         # change year to start from 0 (0 being 2008)
         Year = as.numeric(as.factor(Year)) - 1,
         # dummy variable for grade 5
         Grade5 = ifelse(Grade == "Year 5", 1, 0),
         # dummy variable for grade 7/9
         Grade79 = ifelse(Grade == "Year 7" | Grade == "Year 9", 1, 0),
         # standardised proportion of girls
         P_girls = scale(P_girls),
          # standardised ICSEA
         ICSEA = scale(ICSEA),
         # standardised total enrolments
         N_student = scale(N_student),
         # government vs non-government
         Government = ifelse(Government == "Yes", 1, 0),
         # dummy variable for fixed intercept (moderate exposure)
         Moderate_exposure = ifelse(Exposure_group == "Moderate exposure", 1, 0),
         # dummy variable for fixed intercept (high exposure)
         High_exposure = ifelse(Exposure_group == "High exposure", 1, 0),
         # post mine fire interruption (moderate exposure)
```

```
         Moderate_exposure_fire = Moderate_exposure*Fire,
         # post mine fire interruption (high exposure)
         High_exposure_fire = High_exposure*Fire,
         # post mine fire trend difference (moderate exposure)
         Moderate_exposure_fire_year = Moderate_exposure*Fire*(Year - 6),
         # post mine fire trend difference (high exposure)
         High_exposure_fire_year = High_exposure*Fire*(Year - 6)
         )
```

**Stan model block**

When using Rmarkdown file, stan code can be directly included as a block of code with specification of {stan output.var = "StanModel"} in the code block. In this model we use weakly informative priors, $N(10,5)$, for the SDs of the random school effects, random cohort effects as well as random error $N(10,5)$. 10 was chosen because when using two-level mixed-effects models with the mean score differences as the outcome variable, the estimated error terms are close to 10.

```
data {
  int<lower=0> N; // number of data points
  int<lower=1> C; //number of cohorts
  int<lower=1> J; //number of schools
  int<lower=1, upper=C>  cohortID[N]; //cohort id
  int<lower=1, upper=J>  schoolID[N]; //school id
  int<lower=1> K; // number of predictors
  matrix[N, K] x;   // predictor matrix
  vector[N] y; // outcome
  real<lower=0> sigma[N]; // s.e.'s of outcome
}
parameters {
  real alpha; // intercept

  //SD
  real<lower=0> theta_c; //  for random intercepts for cohorts
```

```
  real<lower=0> theta_s; // for random intercepts for schools
  real<lower=0> theta_t;  // for random error

  //non-centered parameterization
  vector[C] eta_c; //  for random intercepts for cohorts
  vector[J] eta_s; //  for random intercepts for schools
  vector[N] eta_t; //  for random error
  vector[K] beta; // coefficients for predictors
}
transformed parameters {
  vector[C] sigma_c; // random effect of cohort
  vector[J] sigma_s; // random effect of school
  sigma_c = theta_c*eta_c;
  sigma_s = theta_s*eta_s;

}
model {
  eta_c ~ normal(0, 1); // cohort random effects
  eta_s ~ normal(0, 1); // school random effects
  eta_t ~ normal(0, 1); // error

  //prior for SD
  theta_c ~ normal(10, 5); // cohort random effects
  theta_s ~ normal(10, 5); // school random effects
  theta_t ~ normal(10, 5); // random  error

  //prior for coef
  to_vector(beta) ~ normal(0, 50);

  {
  vector[N] mu; // vector of means for all observations
  for (i in 1:N)
    mu[i] = alpha + x[i]*beta + sigma_c[cohortID[i]] +
    sigma_s[schoolID[i]]  + theta_t*eta_t[i];
```

```
    y ~ normal(mu, sigma);
  }
}
```

**Run Stan model**

Input data is needed to be saved in a list.

```
predictors <- c("Year",
              "ICSEA",
              "Grade5",
              "Grade79",
              "P_girls",
              "N_student",
              "Government",
              "Moderate_exposure",
              "High_exposure",
              "Moderate_exposure_fire",
              "High_exposure_fire",
              "Moderate_exposure_fire_year",
              "High_exposure_fire_year")

names <- c( "Year",
          "ICSEA (standardised)",
          "Grade 5",
          "Grade 7 and 9",
          "Proportion of girls (standardised)",
          "Total Enrolments (standardised)",
          "Government",
          "Fixed intercept (moderate exposure)",
          "Fixed intercept (high exposure)",
          "Mine fire interruption effect (moderate exposure)",
          "Mine fire interruption effect (high exposure)",
          "Post-mine fire trend difference (moderate exposure)",
          "Post-mine fire trend difference (high exposure)")
```

```
#Input data stored in a list
input_data <- list(N = nrow(naplan),
                C = length(unique(naplan$Cohort_ID)),
                J = max(naplan$ID),
                cohortID = naplan$Cohort_ID,
                schoolID = naplan$ID,
                K = length(predictors),
                x = naplan[,predictors],
                y = naplan$ScoreDif,
                sigma = naplan$SE)
```

The next stage is to run the simulation using the Stan model defined as 'StanModel'

```
set.seed(12345)
#run stan model
options(mc.cores = parallel::detectCores())
fited_model <- rstan::sampling(StanModel, data = input_data,
          iter = 4000, warmup = 2000,
          control = list(adapt_delta = 0.90))
```

```
fited_model <- readRDS(file = "sampled.Rds")
#saveRDS(fited_model, file = "sampled.Rds")
```

Next we extract results

```
#extract fitted model
extract_model <- rstan::extract(fited_model)
#check mcmc_trace plot
posterior <- as.array(fited_model)

# Save names of SD for random intercepts and errors
thetas <- names(fited_model)[str_detect(names(fited_model),"theta")]
thetas_names <- c("SD of cohort random intercept",
                "SD of school random intercept",
                "SD of test error")
```

```
print(fited_model, pars = c("alpha", thetas,
                            paste0("beta[",1:length(predictors),"]")),
      probs = c(0.5, 0.025, 0.975))
```

```
## Inference for Stan model: 832db374c6d4af44fc1e1f951141a4d4.
## 4 chains, each with iter=4000; warmup=2000; thin=1;
## post-warmup draws per chain=2000, total post-warmup draws=8000.
##
##              mean se_mean   sd    50%   2.5% 97.5% n_eff Rhat
## alpha       -1.32    0.10 4.97  -1.36 -10.99  8.55  2538    1
## theta_c      9.80    0.02 0.79   9.80   8.25 11.33  1755    1
## theta_s     15.87    0.03 1.69  15.80  12.83 19.41  3043    1
## theta_t      2.94    0.05 1.31   3.05   0.28  5.25   711    1
## beta[1]     -0.36    0.00 0.25  -0.36  -0.85  0.12  6474    1
## beta[2]      6.28    0.02 1.14   6.28   4.06  8.49  3814    1
## beta[3]      4.86    0.01 1.01   4.86   2.89  6.82 13630    1
## beta[4]      7.01    0.06 4.38   7.00  -1.60 15.69  5260    1
## beta[5]      2.83    0.01 0.80   2.84   1.26  4.37  9852    1
## beta[6]     -3.79    0.03 2.05  -3.77  -7.82  0.18  3634    1
## beta[7]    -12.89    0.09 4.94 -12.96 -22.55 -3.12  2721    1
## beta[8]     -3.94    0.09 4.52  -4.01 -12.67  5.00  2559    1
## beta[9]    -11.29    0.13 7.10 -11.32 -25.20  2.67  3192    1
## beta[10]    -1.39    0.02 2.05  -1.37  -5.45  2.66  8782    1
## beta[11]   -10.93    0.04 4.06 -10.96 -18.96 -2.82  9621    1
## beta[12]     0.88    0.01 0.73   0.88  -0.56  2.32  8411    1
## beta[13]     2.28    0.02 1.53   2.28  -0.68  5.24  9924    1
##
## Samples were drawn using NUTS(diag_e) at Sun Oct 25 14:46:23 2020.
## For each parameter, n_eff is a crude measure of effective sample size,
## and Rhat is the potential scale reduction factor on split chains (at
## convergence, Rhat=1).
```

Full model diagnostics can be evaluated using an interactive shinystan package. Here we provide a few static diagnostic plots.

```
color_scheme_set("mix-brightblue-gray")
mcmc_trace(posterior,
           pars = c("alpha",thetas,
                    paste0("beta[",1:length(predictors),"]"))) +
  xlab("Post-warmup iteration")
```

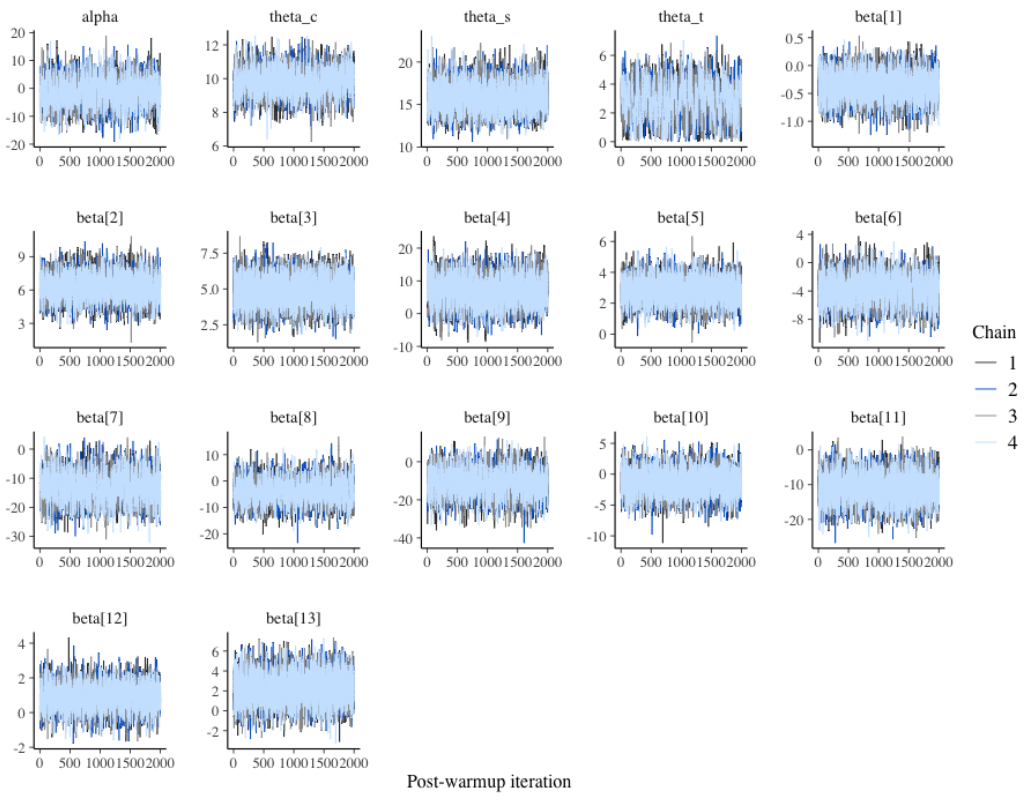


Markov chain Monte Carlo (MCMC) plot shows no signs of poor mixing for each coefficient. There was no warning of divergent transitions (using non-centred parameterisation centered parameterisation can help with avoiding divergent transitions), which can be diagnosed using diagnostic plots for the NUTS. All continuous variables are confounding variables (we are not interested in estimating effect sizes from these parameters), hence they were all standardised in the analysis to improve model fitting speed. If any variable of interest is a continuous variable, the original parameters can be easily recovered (see Stan manual) post standardisation.

The *mcmc_pairs* function is used to visualize the univariate histograms as well as bivariate scatter plots for key parameters. It is useful in identifying multicollinearity (strong correlation) and other non-identifiability issues (banana-like shapes).

```
mcmc_pairs(posterior,
           pars = c("alpha", thetas,
                    paste0("beta[",1:length(predictors),"]"),"lp__"),
           off_diag_args = list(size = 1.5))
```

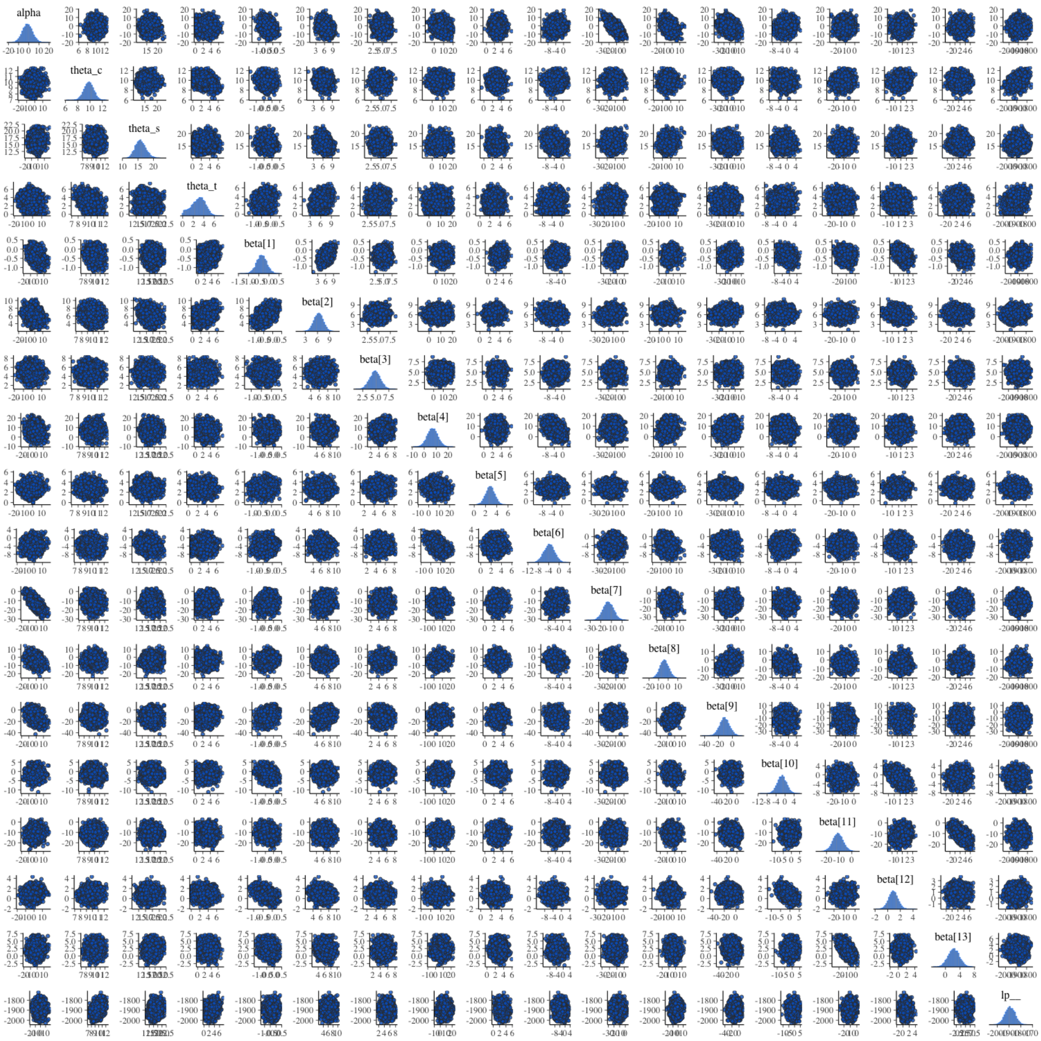


There is a negative association between sampled coefficients of the intercept term (alpha) and school sector (government, beta[7]). This is possible as school sector (government vs non-government ) is the most important predictor of school-level NAPLAN results. Hence the sampled intercept will be impacted by the sampled coefficient of the school sector.

### Model summary

Mean and credible intervals

```
summary <- summary(fited_model,
                   pars = c("alpha",thetas,
                            paste0("beta[",1:length(predictors),"]")),
                   probs = c(0.5, 0.025, 0.975))

names_coef <- c("Intercept", thetas_names, names)

summary <- summary$summary %>%
  as.data.frame() %>%
  mutate(Variable = names_coef ) %>%
  select(Variable, everything())
Summary.Table <- summary %>%
  mutate(Mean = format(round( mean, 2), nsmall = 2),
         `95% CI` = paste0(format(round(`2.5%`,2),nsmall = 2) ,", ",
                           format(round(`97.5%`,2),nsmall = 2))) %>%
  select(Variable, Mean,`95% CI`)

# extract simulated betas and add posterior probability of over or under 0.
B <- data.frame(extract_model$beta)
names(B) <- names
B$ID = rownames(B)
#calculate p over 0 and under 0
p <- B %>%
  as_tibble %>%
  reshape2::melt() %>%
  rename(Variable = variable) %>%
  group_by(Variable) %>%
  summarise(`p (x<0)` = ifelse(mean(value < 0) < 0.001,"<0.001",
                            format(round(mean(value < 0),3),nsmall = 3)) ,
            `p (x>0)` = ifelse(mean(value > 0) < 0.001,"<0.001",
                            format(round(mean(value > 0),3),nsmall = 3))) %>%
```

```
  ungroup()

#merge with result table
Summary.Table <- left_join( Summary.Table, p)

options(knitr.kable.NA = '')
Summary.Table %>%
  kable(align = c("l","c","c","c","c"),booktabs = T,linesep = "") %>%
  kable_styling(bootstrap_options = "striped",
                full_width = F, latex_options = "hold_position")
```

| Variable | Mean | 95% CI | p (x<0) | p (x>0) |
|---|---|---|---|---|
| Intercept | -1.32 | -10.99, 8.55 | | |
| SD of cohort random intercept | 9.80 | 8.25, 11.33 | | |
| SD of school random intercept | 15.87 | 12.83, 19.41 | | |
| SD of test error | 2.94 | 0.28, 5.25 | | |
| Year | -0.36 | -0.85, 0.12 | 0.926 | 0.074 |
| ICSEA (standardised) | 6.28 | 4.06, 8.49 | <0.001 | 1.000 |
| Grade 5 | 4.86 | 2.89, 6.82 | <0.001 | 1.000 |
| Grade 7 and 9 | 7.01 | -1.60, 15.69 | 0.054 | 0.946 |
| Proportion of girls (standardised) | 2.83 | 1.26, 4.37 | <0.001 | 1.000 |
| Total Enrolments (standardised) | -3.79 | -7.82, 0.18 | 0.969 | 0.031 |
| Government | -12.89 | -22.55, -3.12 | 0.994 | 0.006 |
| Fixed intercept (moderate exposure) | -3.94 | -12.67, 5.00 | 0.808 | 0.192 |
| Fixed intercept (high exposure) | -11.29 | -25.20, 2.67 | 0.942 | 0.058 |
| Mine fire interruption effect (moderate exposure) | -1.39 | -5.45, 2.66 | 0.755 | 0.245 |
| Mine fire interruption effect (high exposure) | -10.93 | -18.96, -2.82 | 0.997 | 0.004 |
| Post-mine fire trend difference (moderate exposure) | 0.88 | -0.56, 2.32 | 0.113 | 0.887 |
| Post-mine fire trend difference (high exposure) | 2.28 | -0.68, 5.24 | 0.071 | 0.929 |

### Plot marginal effects

Here we obtain predicted margins using the posterior distribution of coefficients using the following steps:

- Obtain design matrix with confounding variables fixed at reference values
- Calculate posterior distribution of NAPLAN score difference by year and exposure zone
- Plot posterior mean (with error bar) of NAPLAN score difference for each year and exposure zone

```
# create design matrix for calculating marginal effects
Design_matrix <- naplan %>%
   # include the intercept and year variable
   mutate(alpha = 1,Test_year = 2008 + Year) %>%
   select(alpha,predictors,
         Test_year,Exposure_group) %>%
   mutate_at(vars(ICSEA:Government) , function(x) x = mean(x)) %>%
   distinct()


# Obtain contrasts
Matrix <- data.matrix(Design_matrix %>%
                 select(-Test_year, -Exposure_group))
Coef <- t(data.matrix(cbind(extract_model$alpha, extract_model$beta)))
Predicted <- data.frame(Matrix %*% Coef)

names(Predicted) <- paste0("Scores",1:length(Predicted))

# Plot
cbind(Design_matrix[,c("Test_year", "Exposure_group")], Predicted)  %>%
  gather(Measures, scores,-Test_year, -Exposure_group) %>%
  group_by(Test_year, Exposure_group) %>%
  summarise(mean = mean(scores), sd = sd(scores)) %>%
  ggplot( aes(x = Test_year, y = mean,
              group = Exposure_group, col = Exposure_group)) +
  geom_line(size = 1, position = position_dodge(0.4)) +
  geom_errorbar(aes(ymin = mean - sd, ymax = mean + sd),
                position = position_dodge(0.4), size = 0.75) +
  labs(x = "Year",
       y = "Predicted mean NAPLAN score difference",
       col = "Exposure group") +
  geom_vline(xintercept = 2013.66, color = "black",
             size = 2, alpha = 0.3) +
  scale_color_manual(values = c('#fdbb84', '#ef6548','#990000')) +
```

```
scale_x_continuous(limits = c(2008, 2019), breaks = seq(2008, 2018, 1)) +
theme_bw() +
theme(axis.text.x  = element_text(hjust = -0.7,v = -0.1),
      legend.text = element_text(size = 12),
      legend.title = element_text(size = 12),
      axis.title.x = element_text(size = 12,vjust = -0.2),
      axis.title.y = element_text(size = 12),
      legend.position = "bottom")
```

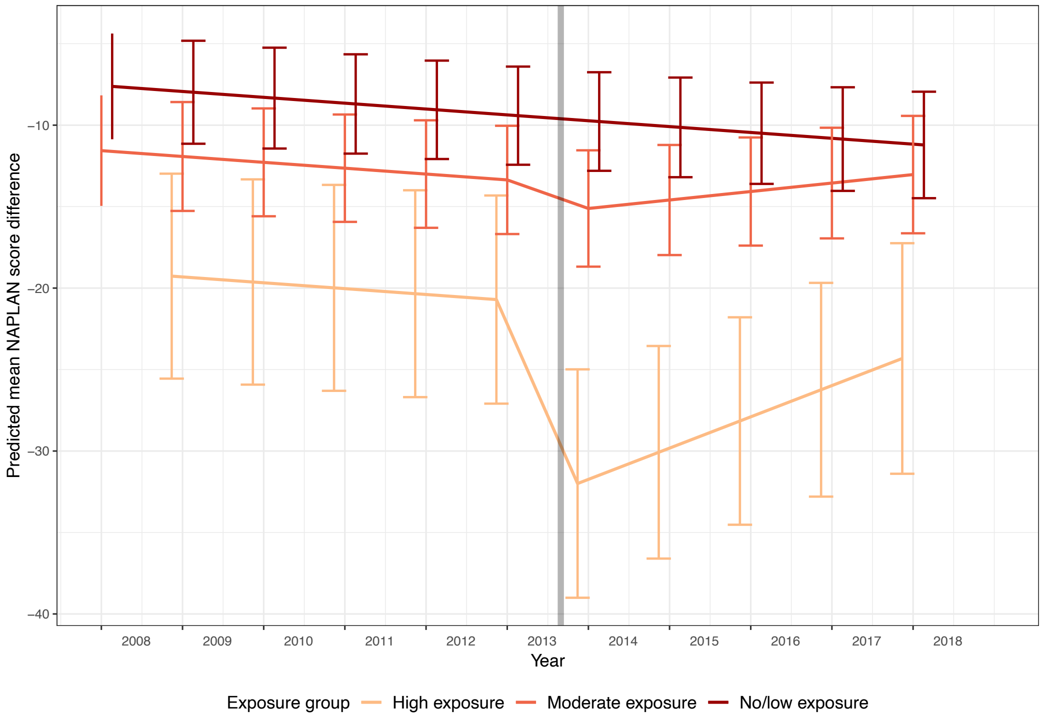

Supplement: S1 File — (PDF) [file pone.0281655.s001.pdf]
