# Supplementary material for: Evaluating the impact of Hazelwood mine fire event on students’ educational development with Bayesian interrupted time-series hierarchical meta-regression
Source: PLoS One. 2023 Mar 1;18(3):e0281655. doi: 10.1371/journal.pone.0281655 (PMC9977026; doi:10.1371/journal.pone.0281655)
Supplement: S2 File — (PDF) [file pone.0281655.s002.pdf]

Supporting Information II - Supplementary tables

Table S1: *Bayesian hierarchical meta-regression models for grammar and punctuation*

	β	95% CI	$P(\beta < 0)$	$P(\beta > 0)$
Intercept	-1.19	-11.15, 8.74		
SD of cohort random intercept	9.71	8.21, 11.25		
SD of school random intercept	15.98	12.92, 19.49		
SD of test error	2.69	0.22, 5.03		
Year	-0.37	-0.85, 0.12		0.07
ICSEA (standardised)	6.12	3.91, 8.30	<0.001	
Grade 5	4.73	2.69, 6.77	<0.001	
Grade 7 and 9	7.22	-1.15, 15.68	0.048	
Proportion of girls (standardised)	2.81	1.21, 4.39	<0.001	
Total enrolments (standardised)	-3.79	-7.73, 0.13		0.029
Government	-12.98	-22.62, -3.34		0.003
Fixed intercept (moderate exposure)	-3.90	-13.13, 5.31		0.20
Fixed intercept (high exposure)	-11.61	-25.28, 2.18		0.05
Mine fire interruption effect (moderate exposure)	-1.78	-5.73, 2.13		0.19
Mine fire interruption effect (high exposure)	-10.91	-18.68, -3.08		0.004
Post-mine fire trend difference (moderate exposure)	0.92	-0.51, 2.35	0.11	
Post-mine fire trend difference (high exposure)	2.37	-0.73, 5.37	0.06	

Table S2: *Bayesian hierarchical meta-regression models for numeracy*

	β	95% CI	$P(\beta < 0)$	$P(\beta > 0)$
Intercept	-2.09	-11.43, 7.13		
SD of cohort random intercept	9.71	8.34, 11.06		
SD of school random intercept	14.67	11.84, 17.86		
SD of test error	5.66	4.17, 7.12		
Year	-0.38	-0.84, 0.07		0.05
ICSEA (standardised)	5.98	3.95, 8.00	<0.001	
Grade 5	3.40	1.69, 5.12	<0.001	
Grade 7 and 9	11.85	3.60, 20.18	0.004	
Proportion of girls (standardised)	1.14	-0.25, 2.52	0.05	
Total enrolments (standardised)	-4.75	-8.43, -0.93		0.008
Government	-7.93	-17.02, 0.92		0.042
Fixed intercept (moderate exposure)	-5.66	-13.94, 2.60		0.08
Fixed intercept (high exposure)	-12.07	-24.26, 0.23		0.028
Mine fire interruption effect (moderate exposure)	-0.44	-4.20, 3.28		0.41
Mine fire interruption effect (high exposure)	-10.90	-17.98, -3.67		<0.001
Post-mine fire trend difference (moderate exposure)	0.79	-0.56, 2.13	0.13	
Post-mine fire trend difference (high exposure)	1.61	-1.21, 4.43	0.13	

Table S3: *Bayesian hierarchical meta-regression models for reading*

	β	95% CI	$P(\beta < 0)$	$P(\beta > 0)$
Intercept	-3.70	-13.03, 5.71		
SD of cohort random intercept	9.47	8.05, 10.90		
SD of school random intercept	15.05	12.05, 18.55		
SD of test error	2.78	0.48, 4.65		
Year	-0.37	-0.82, 0.10		0.06
ICSEA (standardised)	5.83	3.84, 7.93	<0.001	
Grade 5	3.77	1.90, 5.71	<0.001	
Grade 7 and 9	7.66	-0.35, 15.70	0.030	
Proportion of girls (standardised)	1.93	0.48, 3.41	0.005	
Total enrolments (standardised)	-3.77	-7.62, -0.08		0.022
Government	-9.77	-19.02, -0.30		0.022
Fixed intercept (moderate exposure)	-1.87	-10.19, 6.39		0.33
Fixed intercept (high exposure)	-13.41	-26.26, -0.19		0.024
Mine fire interruption effect (moderate exposure)	-1.18	-4.84, 2.57		0.27
Mine fire interruption effect (high exposure)	-8.34	-15.51, -1.07		0.013
Post-mine fire trend difference (moderate exposure)	0.69	-0.61, 2.01	0.15	
Post-mine fire trend difference (high exposure)	1.05	-1.63, 3.85	0.23	

Table S4: *Bayesian hierarchical meta-regression models for spelling*

	β	95% CI	$P(\beta < 0)$	$P(\beta > 0)$
Intercept	-0.79	-9.41, 7.90		
SD of cohort random intercept	10.38	9.19, 11.59		
SD of school random intercept	13.64	11.05, 16.70		
SD of test error	1.31	0.07, 3.04		
Year	-0.48	-0.91, -0.03		0.016
ICSEA (standardised)	4.54	2.63, 6.41	<0.001	
Grade 5	3.62	1.87, 5.37	<0.001	
Grade 7 and 9	5.35	-2.65, 13.79	0.10	
Proportion of girls (standardised)	1.38	-0.07, 2.82	0.030	
Total enrolments (standardised)	-0.94	-4.61, 2.74		0.31
Government	-8.05	-16.52, 0.07		0.026
Fixed intercept (moderate exposure)	-0.52	-8.45, 7.05		0.45
Fixed intercept (high exposure)	-3.68	-16.16, 8.34		0.27
Mine fire interruption effect (moderate exposure)	-1.44	-4.91, 2.09		0.21
Mine fire interruption effect (high exposure)	-10.31	-17.39, -3.38		0.003
Post-mine fire trend difference (moderate exposure)	-0.42	-1.68, 0.85	0.74	
Post-mine fire trend difference (high exposure)	-1.49	-4.20, 1.21	0.86	

Table S5: *Bayesian hierarchical meta-regression models for writing*

	β	95% CI	P($\beta < 0$)	P($\beta > 0$)
Intercept	-1.50	-10.28, 7.31		
SD of cohort random intercept	6.38	4.50, 8.03		
SD of school random intercept	13.94	11.41, 16.87		
SD of test error	7.80	6.36, 9.20		
Year	-0.62	-1.06, -0.16		0.002
ICSEA (standardised)	8.66	6.64, 10.66	<0.001	
Grade 5	3.26	1.35, 5.15	<0.001	
Grade 7 and 9	12.12	4.43, 19.72	<0.001	
Proportion of girls (standardised)	1.57	0.19, 3.00	0.012	
Total enrolments (standardised)	-4.70	-8.38, -0.97		0.006
Government	-13.89	-22.57, -5.43		<0.001
Fixed intercept (moderate exposure)	1.01	-6.83, 8.71		0.61
Fixed intercept (high exposure)	-7.53	-19.63, 4.62		0.11
Mine fire interruption effect (moderate exposure)	3.56	-0.52, 7.55		0.96
Mine fire interruption effect (high exposure)	-11.09	-18.93, -3.16		0.003
Post-mine fire trend difference (moderate exposure)	-0.60	-2.01, 0.82	0.80	
Post-mine fire trend difference (high exposure)	2.44	-0.60, 5.50	0.06	

Table S6: *Estimated intercept, mine fire interruption effect and post-mine fire trend difference for moderate and high exposure schools estimated from Bayesian hierarchical meta-regression models (excluding cohort effect)*

	Moderate exposure				High exposure			
	β	95% CI	P($\beta < 0$)	P($\beta >0$)	β	95% CI	P($\beta < 0$)	P($\beta > 0$)
**Grammar and Punctuation**								
Fixed intercept	-4.27	-12.81, 4.26		0.16	-9.73	-22.73, 3.59		0.07
Mine fire interruption effect	-0.51	-4.71, 3.71		0.40	-13.41	-21.56, -5.17		0.001
Post-mine fire trend difference	0.45	-1.08, 1.94	0.28		2.64	-0.52, 5.78	0.05	
**Numeracy**								
Fixed intercept	-5.55	-13.48, 2.35		0.08	-10.67	-22.94, 1.61		0.043
Mine fire interruption effect	-1.00	-5.02, 2.95		0.31	-13.92	-21.59, -6.39		<0.001
Post-mine fire trend difference	0.71	-0.73, 2.11	0.16		2.35	-0.56, 5.36	0.06	
**Reading**								
Fixed intercept	-2.36	-10.30, 5.79		0.29	-12.06	-23.96, 0.19		0.027
Mine fire interruption effect	-0.77	-4.65, 3.11		0.35	-10.53	-17.84, -2.99		0.002
Post-mine fire trend difference	0.61	-0.78, 1.99	0.19		1.54	-1.30, 4.40	0.14	
**Spelling**								
Fixed intercept	-0.16	-7.79, 7.32		0.48	-0.99	-12.18, 10.33		0.43
Mine fire interruption effect	-2.73	-6.59, 1.12		0.08	-15.79	-23.19, -8.20		<0.001
Post-mine fire trend difference	-0.04	-1.41, 1.31	0.52		-0.18	-3.05, 2.60	0.55	
**Writing**								
Fixed intercept	0.81	-6.87, 8.67		0.58	-7.07	-18.97, 5.12		0.12
Mine fire interruption effect	3.72	-0.10, 7.75		0.97	-13.05	-21.02, -5.16		<0.001
Post-mine fire trend difference	-0.61	-2.04, 0.82	0.80		2.87	-0.21, 5.90	0.034	

Note: All regression coefficients (β), 95% Credible Intervals (95%CI), and posterior probabilities of coefficients under or above 0 [P(β<0), P(β>0)] were estimated from multivariate Bayesian hierarchical meta-regression models, controlling for school-level confounders including ICSEA, total enrolments, percentage of girls, school sector, grade level, long-term. Mine fire interruption effects and post-mine fire trend differences were estimated using the interrupted time-series design

Table S7: *Estimated intercept, mine fire interruption effect and post-mine fire trend difference for moderate and high exposure schools estimated from Bayesian hierarchical meta-regression models (excluding relocated schools)*

	Moderate exposure				High exposure			
	β	95% CI	P(β < 0)	P(β >0)	β	95% CI	P(β < 0)	P(β > 0)
**Grammar and Punctuation**								
Fixed intercept	-3.66	-12.53, 5.03		0.21	-12.03	-27.29, 3.05		0.06
Mine fire interruption effect	-1.96	-5.93, 1.97		0.16	-8.51	-17.39, 0.23		0.027
Post-mine fire trend difference	0.87	-0.53, 2.29	0.12		1.62	-1.61, 4.80	0.16	
**Numeracy**								
Fixed intercept	-5.06	-13.33, 3.40		0.11	-12.22	-26.71, 1.56		0.041
Mine fire interruption effect	-0.70	-4.35, 3.05		0.35	-7.56	-15.76, 0.73		0.036
Post-mine fire trend difference	0.70	-0.64, 2.01	0.15		1.08	-1.95, 4.09	0.24	
**Reading**								
Fixed intercept	-1.77	-10.09, 6.51		0.34	-15.64	-30.60, -1.11		0.018
Mine fire interruption effect	-1.28	-4.88, 2.43		0.24	-5.58	-13.63, 2.43		0.08
Post-mine fire trend difference	0.64	-0.67, 1.96	0.17		1.19	-1.85, 4.19	0.22	
**Spelling**								
Fixed intercept	-0.23	-7.62, 7.54		0.46	-5.96	-19.44, 7.43		0.19
Mine fire interruption effect	-1.65	-5.14, 1.81		0.18	-7.77	-15.87, 0.05		0.026
Post-mine fire trend difference	-0.47	-1.72, 0.76	0.77		-2.09	-5.07, 0.73	0.92	
**Writing**								
Fixed intercept	1.25	-6.57, 9.06		0.62	-9.44	-23.14, 4.23		0.08
Mine fire interruption effect	3.33	-0.49, 7.25		0.96	-9.95	-18.76, -1.17		0.014
Post-mine fire trend difference	-0.70	-2.11, 0.72	0.84		2.30	-1.00, 5.55	0.09	

Note: All regression coefficients (β), 95% Credible Intervals (95%CI), and posterior probabilities of coefficients under or above 0 [P(β<0), P(β>0)] were estimated from multivariate Bayesian hierarchical meta-regression models, controlling for school-level confounders including ICSEA, total enrolments, percentage of girls, school sector, grade level, long-term. Mine fire interruption effects and post-mine fire trend differences were estimated using the interrupted time-series design
