# Supplementary material for: Evaluating the impact of Hazelwood mine fire event on students’ educational development with Bayesian interrupted time-series hierarchical meta-regression
Source: PLoS One. 2023 Mar 1;18(3):e0281655. doi: 10.1371/journal.pone.0281655 (PMC9977026; doi:10.1371/journal.pone.0281655)
Supplement: Attachment — Submitted filename: Review feedback.docx [file pone.0281655.s003.docx]

**Responses to Editor and Reviewer**

**Editor comments**

Apologies for the delay with the peer review. As mentioned it has been difficult to find suitable reviewers for this manuscript. You will find comments from one reviewer which are reasonable and should be addressed in detail. In addition, I have some comments:

**Response**: Thank you for your efforts, we understand the challenges of finding reviewers for a statistical paper, particularly given the impacts of the pandemic. We have addressed the comments in relation to the paper below.

At times the application is referred to as an example of the method being used more generally in climate change work but there is little discussion or comparison of alternative models available for this. As the authors will be aware, there has been some recent literature on interrupted time series design and comparisons made between different approaches (see Turner et al, 2021 as one example). Also, the model used is a fairly simplified version of a time series model in general without seasonality, cyclical effects and autocorrelation being used. This is perhaps fine for this setting, but again if it is being proposed as a method then the simpler case being used could be clearly defined from the general method (and limitations highlighted).

**Response**: Our aim was to develop models to evaluate the impact of climate disasters resulting in major disruptions (see lines 92-100). Models evaluating climate change impacts that are gradual (e.g., increases in temperature and air pollution level) were not considered here. We have removed the climate change statement in the abstract as suggested by the reviewer.

The primary method of studying the impact of climate disasters in the research literature is still via individual-level surveys. There is a merging area focusing on using secondary data [1, 2]. However, when only aggregated data is available, the time series design is perhaps more accurate compared with other methods such as simple pre and post-comparison or difference-in-difference.

As the editor has indicated, there are many methods to estimate an interrupted time series model. A series of papers by our Monash University colleagues Simon Turner and others have provided an excellent summary and evaluation of existing interrupted time series methods [3, 4]. However, their work primarily focused on interrupted time series models for one outcome in individual series. Our study, on the other hand, has two outcome parameters (mean and SE) and multiple series, so these methods are not directly comparable. It is possible to estimate our model using a linear fixed-effects or mixed-effects model using a frequentist regime, which we are testing at the moment with a large number of schools included, in which the Bayesian models can be very costly to estimate. We have included this in the discussion part, see lines 347-439.

*“Similar models can be fitted via a frequentist regime using mixed-effect meta-regression models (e.g., estimated using restricted maximum likelihood). Although frequentist models do not take advantage of prior distributions, they generally yield similar results to Bayesian models [5]. “*

The NAPLAN test is only conducted once a year at a fixed time (mid-may), so the seasonality and cyclical effects are largely not relevant in this setting (we have made this clear in the manuscript). The autocorrelation is potentially an issue in modelling NAPLAN scores, however, it is theoretically unlikely because the students being assessed in two consecutive years are different (NAPLAN tests are only conducted in grades 3, 5,7 and 9, not every grade). The same group of students were assessed every two years, which is modelled by the random intercepts of waves of students. The main limitations of our model are that random slopes and non-linear long-term trends are not modelled. As there were only 69 schools included, more complicated models may not be generalisable even if the model can be fitted via the sampling framework. These were discussed in lines 4654-469.

*“More complicated models such as random slopes, non-linear long-term trends and autocorrelation structure, were not considered due to the unwarranted increase in modelling complexity with limited data. These models can be applied when there are more schools and time points available.”*

The factors responsible for the decline in academic achievement are not convincingly discussed given the countervailing forces involved. For example, is there any information available about levels of funding to support the schools affected and specific interventions that were made? The results then seem to be in spite of these interventions being available (and perhaps were not effective in some areas?). Forces influencing the quality of teaching don’t seem to be discussed but could relate to teachers leaving the area, staff turnover, trauma experienced by the teachers, etc. Other factors not measured/available in the model also could be discussed in terms of changes to curriculum/schooling, any other events in the area around this time which could be involved. Some of this could be speculative but gives some insight into the factors affecting the quality of education for schools in particular areas. This could also be part of the translation public health/educational messaging in the Discussion section which is reasonably weak at the moment in terms of this.

**Response**: We have updated our discussion to include more details as suggested, e.g., lines 362-373, lines 385-394. The psychological trauma for both students and teachers and interruptions to school functioning are believed to be the primary driving force behind the decline. There was no immediate funding available post the mine fire, however, additional funding was allocated in later years to upgrade school facilities. While we are anecdotally aware of post-disaster programs being put in place following the mine fire, we have been unable to access any relevant information regarding this. Curriculum/schooling remains largely unchanged in the area.

The influence of the two relocated schools seems to be important in terms of the results (Table S7) but the discussion on this seems to be only a sentence in the sensitivity analysis. Thus, there is room to discuss the influence of this with more insight as to the overall results. This is important given the Discussion on possible reasons for the decline in scores.

**Response**: Thank you for pointing this out. We agree that these can be important for policy guidance. We have included additional discussion around this point, see lines 362-373.

*“The mine fire also introduced disruption to day-to-day operations in nearby schools [6]. Two schools were relocated causing direct educational interruptions as well as threats to students’ sense of safety and security [6]. The models excluding these two schools showed a slightly lower level of interruption in high exposure schools compared with when they were included. This suggests that school relocation might have had an adverse impact on academic performance in addition to the mine fire exposure. Prior research has found that school relocation has a negative effect on students as well as teachers. Students are impacted by the change of routine, additional time traveling to new school locations, and uncertainty about the permanency of school relocation. Teachers experience similar uncertainty and concern about the effects of school relocation on students, and have reported that school relocation increases their workload and decreases time spent in the classroom with children [6]. These impacts should be considered during disaster risk reduction planning and policies of schools.”*

I couldn’t see a clear definition for what was considered pre and post time periods in terms of NAPLAN scores (e.g when NAPLAN was carried out). If it is there make it clearer. Relating to this is the sensitivity of the results in terms of the timing of NAPLAN results and definitions of pre and post mine periods? NAPLAN results may not an immediate indicator for that year but rather of previous learning so there could/will be a time delay involved.

**Response**: NAPLAN is held annually in May, and this was previously described in line 142 (now line 174). We collected NAPLAN data for the years 2008-2018 as previously shown in line 149 (now 181). We have now added information to define the pre- (2008-2013) and post-mine fire period (2014-2018) in lines 190-191. NAPLAN testing is designed to track the academic progression of students over time. There are a few factors relating to the mine fire event which may have impacted NAPLAN results: (1) trauma and stress directly impacting students’ cognitive ability which can be immediately observed, (2) interruptions in the delivery of educational activities which can also be observed in the NAPLAN test 3 months after the mine fire, (3) long-term changes for the socioeconomic status that can be observed in a few years following the mine fire. From the results, it seems that most schools were able to gradually recover, therefore it is likely that the lag effect is not strong.

**Reviewers 1 comments:**

The authors present a method to use available aggregated data on students performance to estimate the impact of a significant environment event (mine fire), applying Bayesian hierarchical meta-regressions and an interrupted time series design. The study is well conducted, its methodology is well presented and supported, and the results are consistent with some previous research on the topic. The study is an important contribution to the scientific community and the manuscript can be published after addressing the aspects described below:

**Response**: Thank you for these positive comments. We have addressed the comments in relation to the paper below.

Abstract: Line 30. “With the increasing threat of climate change” this does not link to what is provided in the methods and results.

**Response**: Thank you for alerting us to this. This text has been removed.

Line 31. “a new analytical framework to evaluate the impact of climate disasters”. This is a very comprehensive (and ambitious) objective. Also, this was not addressed this way in the methods, results and discussion sections.

**Response**: We have changed this to “develop a Bayesian model using aggregated school-level data to evaluate the impact of climate disasters on academic achievement”, now lines 30-31.

Line 34. The word “only” can be confusing since the models include several co-variates. The authors might find a better way to highlight the advantage of the proposed methodology to use this kind of easily available data.

**Response**: The term “only” has been replaced with “easily accessible”, now line 35

Line 44. Conclusion or discussion?

**Response**: This heading has been changed to conclusion, now line 45.

Introduction: Multiple citations are from old studies. Strongly suggest considering more recent references. Lines 53, 57, 59. Suggest having more recent citations. Line 65. Consider more recent research to support the background/introduction

**Response**: We have updated most of the references as suggested, except for a few seminal papers (e.g., Norris et al., 2002 and McFarlane et al., 1987).

Parag. Starting in line 66. Some redundancy with previous paragraph. Maybe the 2 parag. can be merged.

**Response**: We agree with this suggestion. These paragraphs have now been merged, see lines 67-76.

Line 85. This statement must be supported by more recent research

**Response**: We have modified the sentence and included more recent publications, see lines 89-91.

*“However, the psychological and academic needs of children and adolescents can be overlooked or insufficiently responded to after disasters [7-10].”*

Paragraphs from line 100-114 are somewhat disconnected from the last statement in line 99 that should link to the 2nd and 3rd sentences in the last paragraph of the introduction. This is the focus of the paper while the Hazelwood event is used as a case study.

**Response**: We agree that a linking statement is needed. We have included the following additional sentence at the start of the previous paragraph, now lines 106-107.

*“In this study, we proposed a new method to address these challenges by examining the impact of the Hazelwood mine fire.”*

Methods Line 124-125. You can remove “the town”

**Response**: Thank you, this has now been removed.

Lines 127-131. Suggest some pre-explanation of the area levels (it’s a bit confusing. Not all readers would be familiar with these Australian areas).

**Response**: The following information has now been provided in the manuscript, lines 134-138.

*“The school-level exposures were assigned based on Australian Statistical Geography Standard (ASGS) boundaries [11]. ASGS divides geographical areas, also known as statistical areas (SA), into four levels, including: SA1 - the smallest census unit with a population between 200-800; SA2 - comparable to township; SA3 comparable to a local government area; and SA4 -largest sub-State regions.”*

First para. Further explanation needed to understand why and how the modelled PM25 was used to specify the selection of schools.

**Response**: The following information on how the PM_2.5_ data were modelled and processed has now been provided, lines 139-145.

*“Due to a lack of air quality monitoring data in the initial mine fire period when pollution levels were highest [53, 54], retrospective modelling of air pollution levels was conducted by CSIRO [55]. Hourly emissions of mine fire-related particulate matter exposure (PM_2.5_) were estimated using a prognostic meteorological and pollutant dispersion model at a resolution of 1km x 1km, see details published elsewhere [55]. The estimated spatial-temporal PM_2.5_ concentrations were aggregated by SA2 to understand the geographical distribution of cumulative mine fire-related air pollution exposure.”*

Line 132. Not sure of the meaning (need) of the last part of this sentence.

**Response**: The air pollution model provides a high-resolution spatial and temporal estimate of mine fire PM_2.5_ exposure, so the exposure at the location of each school can be estimated (either using exposure level in the 1km x 1km grids estimated directly from the air pollution model or the aggregated SA2-level exposure). However, the student also had exposure at their residential home address which may or may not be close to their schools, and which we were not able to estimate given we were restricted to aggregate data. So, we divided schools into three exposure groups (high, moderate and no/low exposure) rather than using the estimated PM_2.5_ level at the individual school location as a continuous exposure variable. We have modified this paragraph to make it clear.

*“The modelled mine fire-related PM_2.5_ concentrations suggested that the Morwell SA2 closest to the mine fire experienced the highest smoke concentrations, which at times greatly exceeded national safety standards [12]. Prevailing winds largely restricted the dispersion of the smoke plume to the Latrobe Valley SA3 region (which includes Morwell), and that area is considered to have suffered a moderate level of exposure [12]. Further to the east, the SA3 area of Wellington Shire, with a socioeconomic profile similar to that of Latrobe Valley, had little to no exposure to the smoke. Therefore, Wellington Shire was chosen as the comparison area for this analysis. There were 69 primary and secondary schools in the region which were classified into three mine fire exposure groups: Morwell (high exposure), the remainder of the Latrobe Valley (moderate exposure) and Wellington (no/low exposure), see Fig 1.”*

Line 151. Some explanation on the interpretation of the ICSEA is needed (higher or lower scores indicate more SE advantage?)

**Response**: Thank you, this has now been included in the manuscript, lines 183-187.

“ICSEA is a scale indicating socioeducational advantage of the school based on students’ family background information, which ranges from about 500 (extremely disadvantaged student backgrounds) to about 1300 (extremely advantaged student backgrounds)."

Figure 1 would be benefited if the location of the fire is shown (or another figure so the spatial setting is better contextualised regarding the exposure source). Why areas west of Morwell are not considered? (maybe this links to the need of better explanation of the PM25 modelling)

**Response**: The fire location is now marked in Fig 1. Other than the SA2s immediately to the west of Morwell (which are included in the Moderate Exposure group in Fig 1), areas further to the west are not considered for two reasons: the prevailing winds kept the smoke plume largely restricted to the Latrobe Valley area, and areas further west of Morwell typically have a higher socioeconomic status. The Latrobe Valley is an area with a relatively low socioeconomic status, which is more comparable to Wellington Shire to the east of the Latrobe Valley. This information was provided in lines 146-155.

Line 169:

**Response**: Thank you, this has now been corrected, now line 203.

Line 170. Any previous research using this approach?

**Response**: No. We haven’t been able to find any similar studies. Almost all studies use individual-level academic scores. There are a few new studies used school-level scores, but only the mean scores were used, hence our argument that this statistical approach is a valuable addition to the field.

Good inclusion of figure 2

**Response**: Thank you.

Statistical analysis is well introduced and supported

**Response**: Thank you.

Line 228. Some description of the priors tested in the sensitivity analyses and the main outcomes of excluding cohort RE should be included in the supp. Material

**Response**: The sensitivity analysis of excluding cohort RE was included in Table S6 in the Supplementary Material. However, the sensitivity analysis using different priors was not because we have tested a few choices, e.g., non-informative priors, weakly informative priors for random effects and non-informative priors for coefficients, and weakly informative priors for all parameters. As a result, it was challenging to include all of these sensitivity analyses. As we have provided source data (simulated), interested users can test different models using the data provided.

Results: Table 1 presents 2 different measures in parentheses -this needs to be better clarified in the table title.

**Response:** We have changed the notes to better describe the statistics reported.

*“Statistics presented are either the median (IQR: Q1, Q3) or sample size n (%)”*

Line 262. Review these figures

**Response**: The exact range was -3.68 to -13.41. We approximate by the lower and upper bound, which is -3 and -14. We have now changed the numbers to represent the exact range.

Lines 264-27 data already shown in table 2

**Response**: We have simplified the sentence as:

*“Table 2 also shows that there were substantial interruption effects post-mine fire in high exposure schools across all academic domains, with estimated mean score reductions of between* 10.31 and 11.09 *for grammar and punctuation, numeracy, spelling and writing, and 8.34 for reading.”*

Paragraph starting in line 290. This answers one of my comments above.

**Response**: Thank you, change made.

Discussion Line 303. “The results suggest..”

**Response**: Thank you, change made.

Line 305. Suggest” “the detrimental impact…” (avoid confusion —impacts can also be positive)

**Response**: Thank you, change made.

Line 326. “The potential links…”

**Response**: Thank you, change made.

Line 331. “the Hazelwood mine fire smoke”

**Response**: Thank you, change made.

Line 363. “also” seems redundant here

**Response**: Thank you, “also” has been deleted.

Line 366. Previous section did not clarify specifically how the analysis address the spatial profile of the impacts (models do not include a spatial term?)

**Response**: The spatial profile refers to the geographically defined exposure zones. We have included the following text to make this clear.

*“Firstly, it enables the evaluation of the spatial (no/low exposure to high exposure areas) and temporal profile of the impact, which could be used to inform policy and resource allocation in academic settings after disasters.”*

The discussion needs a section to discuss the method used in this study—the strengths section somewhat addresses this but there is not a formal discussion of the specifics of the methods and comparison with other research

**Response**: We have included a separate section on statistical modelling approach and moved relevant information into this section, see lines 411-439.

The limitations section should address potential ecological bias and how this might affect the results and their interpretation. Also how the limitations already commented in this section might impact the findings and how this could be controlled in further research.

**Response:** We agree that the ecological bias may impact our findings. Therefore, we have expanded our discussion around this, as follows:

*“Furthermore, the aggregated nature of the data prohibited* controlling for individual-level risk factors*. While the interrupted time series design was used to allow post-mine fire scores in individual schools to be compared relative to their past, there may be other changes in students’ profiles over time, which may impact our findings. For example, some families may have left the region due to the subsequent closure of the mine and power station. Advanced population-level data linkage methods can be used to explore these issues. However, as noted above, accessing population-level data can be challenging and time-consuming.”*

Conclusions: Suggest naming the fire event specifically in the first line

**Response**: Updated, thank you.

Last line would be better commented in the discussion not in this section

**Response**: The last sentence was moved to the discussion part on the method.

**Journal requirements:**

When submitting your revision, we need you to address these additional requirements.

1. Please ensure that your manuscript meets PLOS ONE's style requirements, including those for file naming.

**Response**: We have updated the manuscript according to the PLOS ONE's style requirements

2. Please provide additional details regarding participant consent. In the ethics statement in the Methods and online submission information, please ensure that you have specified (1) whether consent was informed and (2) what type you obtained (for instance, written or verbal, and if verbal, how it was documented and witnessed). If your study included minors, state whether you obtained consent from parents or guardians. If the need for consent was waived by the ethics committee, please include this information. If you are reporting a retrospective study of medical records or archived samples, please ensure that you have discussed whether all data were fully anonymized before you accessed them and/or whether the IRB or ethics committee waived the requirement for informed consent. If patients provided informed written consent to have data from their medical records used in research, please include this information.

**Response**: Only aggregated school-level public data was used therefore individual-level consent was not needed.

3. Thank you for stating the following financial disclosure: “This work was funded by the Victorian Department of Health and Human Services.” Please state what role the funders took in the study.  If the funders had no role, please state: ""The funders had no role in study design, data collection and analysis, decision to publish, or preparation of the manuscript." If this statement is not correct you must amend it as needed. Please include this amended Role of Funder statement in your cover letter; we will change the online submission form on your behalf.

**Response**: While the overarching design for the wider Hazelwood Health Study was one of the factors assessed by the funders in the original awarding of the research contract, the funders had no role in the specific design, data collection or analysis of this research component. While the funders did not play a role in the decision to publish or the preparation of the manuscript, they were provided with the opportunity to review and approve the manuscript before submission. As part of this review process, the funders made minor suggestions regarding inconsistent language which were corrected. There were no changes made to the analysis and interpretation as a result of this feedback.

4. Thank you for stating the following in your Competing Interests section:  “NO authors have competing interests.” Please complete your Competing Interests on the online submission form to state any Competing Interests. If you have no competing interests, please state ""The authors have declared that no competing interests exist."", as detailed online in our guide for authors at <http://journals.plos.org/plosone/s/submit-now> This information should be included in your cover letter; we will change the online submission form on your behalf.

**Response**: please see this information in the cover letter.

5. We noted in your submission details that a portion of your manuscript may have been presented or published elsewhere. [The manuscript is published on the preprint server <https://www.medrxiv.org/content/10.1101/2021.03.28.21254516v2>] Please clarify whether this [conference proceeding or publication] was peer-reviewed and formally published. If this work was previously peer-reviewed and published, in the cover letter please provide the reason that this work does not constitute dual publication and should be included in the current manuscript.

**Response**: This manuscript identified above is a pre-print version of this article which was not peer-reviewed. This information was provided in the original cover letter and we have included this again as requested.

6. In your Data Availability statement, you have not specified where the minimal data set underlying the results described in your manuscript can be found. PLOS defines a study's minimal data set as the underlying data used to reach the conclusions drawn in the manuscript and any additional data required to replicate the reported study findings in their entirety. All PLOS journals require that the minimal data set be made fully available. For more information about our data policy, please see <http://journals.plos.org/plosone/s/data-availability>. Upon re-submitting your revised manuscript, please upload your study’s minimal underlying data set as either Supporting Information files or to a stable, public repository and include the relevant URLs, DOIs, or accession numbers within your revised cover letter. For a list of acceptable repositories, please see <http://journals.plos.org/plosone/s/data-availability#loc-recommended-repositories>. Any potentially identifying patient information must be fully anonymized. Important: If there are ethical or legal restrictions to sharing your data publicly, please explain these restrictions in detail. Please see our guidelines for more information on what we consider unacceptable restrictions to publicly sharing data: <http://journals.plos.org/plosone/s/data-availability#loc-unacceptable-data-access-restrictions>. Note that it is not acceptable for the authors to be the sole named individuals responsible for ensuring data access. We will update your Data Availability statement to reflect the information you provide in your cover letter.

**Response**: The data used in this study belonged to a third party, the Australian Curriculum, Assessment and Reporting Authority (ACARA) and access to the data requires permission from the ACARA data custodians. As such, we are unable to make a minimal data set available. Instead, a synthetically generated data set based on the source data for the tutorial part of the paper has been made available at <https://doi.org/10.5281/zenodo.6613107>

7. We note that you have indicated that data from this study are available upon request. PLOS only allows data to be available upon request if there are legal or ethical restrictions on sharing data publicly. For more information on unacceptable data access restrictions, please see <http://journals.plos.org/plosone/s/data-availability#loc-unacceptable-data-access-restrictions>. In your revised cover letter, please address the following prompts: a) If there are ethical or legal restrictions on sharing a de-identified data set, please explain them in detail (e.g., data contain potentially sensitive information, data are owned by a third-party organization, etc.) and who has imposed them (e.g., an ethics committee). Please also provide contact information for a data access committee, ethics committee, or other institutional body to which data requests may be sent. b) If there are no restrictions, please upload the minimal anonymized data set necessary to replicate your study findings as either Supporting Information files or to a stable, public repository and provide us with the relevant URLs, DOIs, or accession numbers. For a list of acceptable repositories, please see <http://journals.plos.org/plosone/s/data-availability#loc-recommended-repositories>.We will update your Data Availability statement on your behalf to reflect the information you provide.

**Response**: The data used in this study belong to ACARA and access to the data requires permission from the ACARA data custodians. We have obtained the DOI for the release of the synthetically generated data.

8.  Your ethics statement should only appear in the Methods section of your manuscript. If your ethics statement is written in any section besides the Methods, please delete it from any other section.

**Response**: Thank you, we have moved the ethics statement to the Methods section.

**References**

1.	Gibbs L, Nursey J, Cook J, Ireton G, Alkemade N, Roberts M, et al. Delayed Disaster Impacts on Academic Performance of Primary School Children. Child Development. 2019;90(4):1402-12. doi: <https://doi.org/10.1007/s12310-016-9175-2>.

2.	Reifels L, Bassilios B, Spittal MJ, King K, Fletcher J, Pirkis J. Patterns and Predictors of Primary Mental Health Service Use Following Bushfire and Flood Disasters. Disaster Medicine and Public Health Preparedness. 2015;9(3):275-82. Epub 2015/04/14. doi: 10.1017/dmp.2015.23.

3.	Turner SL, Forbes AB, Karahalios A, Taljaard M, McKenzie JE. Evaluation of statistical methods used in the analysis of interrupted time series studies: a simulation study. BMC Medical Research Methodology. 2021;21(1):181. doi: 10.1186/s12874-021-01364-0.

4.	Turner SL, Karahalios A, Forbes AB, Taljaard M, Grimshaw JM, Cheng AC, et al. Design characteristics and statistical methods used in interrupted time series studies evaluating public health interventions: a review. J Clin Epidemiol. 2020;122:1-11. Epub 20200225. doi: 10.1016/j.jclinepi.2020.02.006.

5.	Tanriver-Ayder E, Faes C, van de Casteele T, McCann SK, Macleod MR. Comparison of commonly used methods in random effects meta-analysis: application to preclinical data in drug discovery research. BMJ Open Science. 2021;5(1):e100074. doi: <https://doi.org/10.1136/bmjos-2020-100074>.

6.	Berger E, Carroll M, Maybery D, Harrison D. Disaster Impacts on Students and Staff from a Specialist, Trauma-Informed Australian School. Journal of Child & Adolescent Trauma. 2018;11:1-10. doi: <https://doi.org/10.1007/s40653-018-0228-6>.

7.	McFarlane AC, Policansky SK, Irwin C. A longitudinal study of the psychological morbidity in children due to a natural disaster. Psychological Medicine. 1987;17(3):727-38. Epub 2009/07/09. doi: <https://doi.org/10.1017/S0033291700025964>.

8.	Silverman WK, La Greca AM. Children experiencing disasters: Definitions, reactions, and predictors of outcomes. 2002:11-33. doi: 10.1037/10454-001.

9.	Seddighi H, Salmani I, Javadi MH, Seddighi S. Child Abuse in Natural Disasters and Conflicts: A Systematic Review. Trauma, Violence, & Abuse. 2019;22(1):176-85. doi: 10.1177/1524838019835973.

10.	Griffith AK. Parental Burnout and Child Maltreatment During the COVID-19 Pandemic. Journal of Family Violence. 2022;37(5):725-31. doi: 10.1007/s10896-020-00172-2.

11.	ABS. Australian Statistical Geography Standard (ASGS): Australian Bureau of Statistics (ABS); 2011. Available from: <https://www.abs.gov.au/websitedbs/d3310114.nsf/home/australian+statistical+geography+standard+(asgs>).

12.	Luhar AK, Emmerson KM, Reisen F, Williamson GJ, Cope ME. Modelling smoke distribution in the vicinity of a large and prolonged fire from an open-cut coal mine. Atmospheric Environment. 2020;229:117471. doi: <https://doi.org/10.1016/j.atmosenv.2020.117471>.
