# Supplementary material for: Evaluating the impact of Hazelwood mine fire event on students’ educational development with Bayesian interrupted time-series hierarchical meta-regression
Source: PLoS One. 2023 Mar 1;18(3):e0281655. doi: 10.1371/journal.pone.0281655 (PMC9977026; doi:10.1371/journal.pone.0281655)
Supplement: Attachment — Submitted filename: Responses to Reviewers R2.docx [file pone.0281655.s004.docx]

**Responses to Reviewers**

Our sincere thanks to the reviewers for providing such detailed and helpful comments on our manuscript. We have responded to each comment below and highlighted relevant changes in the revised manuscript.

**Reviewer #1:**

The authors have rightly addressed most of my previous comments in the first version of the manuscript. This allowed us to have a better appreciation of the analysis and discussion from which some further comments and suggestions should be addressed before acceptance for publication. Please see specific comments for each section below.

**Response**: Thank you for these positive comments. We have addressed each of the reviewer’s comments in relation to the paper below.

Line 29. Maybe you meant “publicly available” rather than easily?

**Response**: Data for individual schools is available online (https://www.myschool.edu.au/). To obtain all data, researchers can apply for data access via the Australian Curriculum Assessment and Reporting Authority (ACARA), which assesses research applications on the basis of merit. Therefore, we feel that “easily accessible” is still slightly better in describing the accessibility of the data.

Lines 59-61. Maybe “environmental disasters” is a better context than “climate disasters” -or a better contextualisation on how this industrial disaster matches a climate event is needed. This would align with the citations used as some relate to natural disasters. Lines 70, 79. Consider the above comment.

**Response**: We agree with the reviewer’s perspective that “environmental disasters” is a broader and better term to use in our paper. We have updated accordingly throughout the document.

Lines 100-101. This links to a lack of research on health impacts of the coal mining industry in Australia, as health aspects are quite related to cognitive development and performance -this can be addressed in the discussion.

**Response**: Thanks for pointing this out. We have added additional discussions in lines 355-361.

***“Fire incidents are common in coal mines [70] and, all too often, mining project impact assessments adopt a narrow range of health and wellbeing domains that omit consideration of potential social consequences [71]. Accordingly, impacts that events such as the Hazelwood mine fire can have on academic outcomes among school-aged young people warrant consideration within mine project planning and, more generally, within disaster risk reduction planning and education department policies.”***

Line 110. Suggest to add specific context of populations affected (i.e., children in the proximity, etc..)

**Response**: The indicator of geospatial location was included (lines 101-102; inserted text in bold below) and we have also referenced the air pollution study for further details.

*“Whilst the flames themselves did not directly threaten homes or lives, heavy smoke concentrations throughout the six-week period (****particularly in the nearby town of Morwell and the wider Latrobe valley area) [42]*** *resulted in increased mortality, physical ill-health and psychological distress in the local community [35, 43-47].”*

Methods Line 175. Not sure these coefficients are “comparable” to individual level analyses.

**Response**: The results from Bayesian models using school-level distributions should obtain similar results to modelling outcomes based on individual students’ records, as long as the assumptions of the Bayesian models can be met. While there is a clear benefit in using individual-level data because it allows individual-level factors (e.g., age, gender, adverse childhood events, parent educational status) to be considered, accessing such data can be extremely challenging. Although we took some of these factors into consideration in the model (e.g., school type, percentages of female students, area-based socioeconomic status), not including individual-level data may still have an impact on the results. In this study, it is unlikely that some of these factors caused differences in exposure (i.e. adverse childhood events will not cause a higher or lower mine fire exposure level). However, we agree that it is important to make the claim clear. We have modified the text to be more specific, see line 183.

*“This approach directly models distributions of students’ performance within schools using mean scores and standard errors, which provides unbiased estimates that are* ***broadly*** *comparable with the model using individual-level records (****not considering individual-level confounding factors****).”*

Methods: Line 55. Much recent citations can back this statement

**Response**: Thank you. We have included the updated CRED report and the most recent IPCC report.

*“The frequency and severity of disasters such as wildfires have increased dramatically in recent years within the context of climate change [2-4].”*

*“2. Xu R, Yu P, Abramson MJ, Johnston FH, Samet JM, Bell ML, et al. Wildfires, Global Climate Change, and Human Health. New England Journal of Medicine. 2020;383(22):2173-81. doi:* [*https://doi.org/10.1056/NEJMsr2028985*](https://doi.org/10.1056/NEJMsr2028985) *.*

*3.	IPCC. Summary for Policymakers. In: Climate Change 2021: The Physical Science Basis. Contribution of Working Group I to the Sixth Assessment Report of the Intergovernmental Panel on Climate Change. 2022. Available from:* [*https://www.ipcc.ch/report/ar6/wg1/#SPM*](https://www.ipcc.ch/report/ar6/wg1/#SPM) *.*

*4.	CRED. The Non-Covid year in disasters: Global trends and perspectives. Centre for Research on the Epidemiology of Disasters (CRED), UN Office for Disaster Risk Reduction., 2021. Available from:* [*http://hdl.handle.net/2078.1/245181*](http://hdl.handle.net/2078.1/245181)*.”*

Methods: Line 206. How does this equation link to the model shown in line 192 (I don’t remember if this was asked in the first review -maybe some of the explanations from lines 207-216 can be better introduced with equations linked to the regression model).

**Response**: It is slightly unclear to us whether “Line 206” refers to the track-changed document or no track-changed document. The question makes sense in both these two places.

If “Line 206” refers to the equation (1) in the document without track-change, we included additional information that equation (2) is the expansion of the interrupted time series terms in equation (1) in lines 215-216:

***“The interrupted time series terms in*** $\boldsymbol{X}_{\boldsymbol{s,g}}\boldsymbol{\beta}$ ***in Equation (1) are summarised as follows”***

However, “Line 206” can also refer to “Interrupted time series models using only means with linear regression instead of meta-regression will be inefficient (significant loss of statistical power) and biased towards smaller schools (large schools with more students should be given more weights)” in the document with track-change. If this is the case, we have included additional information regarding weights in lines 183-187.

*“Interrupted time series models using only means with linear regression instead of meta-regression will be inefficient (significant loss of statistical power) and biased towards smaller schools (large schools with more students should be given more higher weights, e.g.,* ***using*** ***regression models weighted by the number of students or meta-regressions with inverse variance as weights****).”*

Results: Line 267. Maybe you meant “i.e.,,” rather than “e.g.,”

**Response**: This is now corrected (see line 285). Thank you.

Discussion: Line 313. Remove “some”

**Response**: This has been deleted (see line 331).

Discussion: Lines 337-338. The authors should expand more on poor impact assessment in the coal mining sector. Very few but still some important studies relevant to Australia would support this (consider: doi.org/ 10.1093/heapro/daz032 and doi.org/ 10.1515/reveh-2019-0033), see lines 355-361.

**Response**: We have added an assertion within the discussion section in relation to the reviewers’ suggestion here, and have made use of one of the references forwarded.

***“Fire incidents are common in coal mines [70] and, all too often, mining project impact assessments adopt a narrow range of health and wellbeing domains that omit consideration of potential social consequences [71]. Accordingly, impacts that events such as the Hazelwood mine fire can have on academic outcomes among school-aged young people warrant consideration within mine project planning and, more generally, within disaster risk reduction planning and education department policies.”***

Discussion: Lines 346-348. From this line you should reconsider the following statement in the first parag of the discussion […] the …“mine fire had a major detrimental impact on academic performance”

**Response**: Thank you for pointing this out. We use “delineate the relative contributions” to refer to the fact that there are a few possible mechanisms of how mine fire exposure may contribute to the overall detrimental impact. Therefore, further investigation is needed to understand which mechanism is more important and to develop targeted interventions. We have modified the text to make this clear, see lines 365-376.

*“****Although our study has shown a clear impact of the mine fire on academic performance, the model cannot delineate between the potential underlying mechanisms related to the exposure, such as psychological trauma, disruption to schooling, or air pollution exposure.*** ***Accordingly, further investigation is needed to understand the relative contributions of different mechanisms by which disaster exposure impacts academic performance, which could inform the development of targeted interventions.****”*

Discussion: Section “comparison with other studies”. I suggest the authors also address the differences in impacts from different “types” of disaster. Natural disasters most probably have a different reaction/effect at the community level than disaster in industries with big economic involvement -as there is some duty of care related to the lack of prevision (consider the impact assessment suggestions above).

**Response**: While there is a dearth of literature regarding whether academic performance is differentially impacted by whether disasters are natural or human-caused, there is evidence to suggest that other health outcomes such as the lifetime risk of psychiatric disorders are more associated with human-caused events [1]. However, it is slightly difficult to classify Hazelwood mine fire in the spectrum of “natural” to “man-made” disasters. The mine fire was initiated by a bushfire. However, a significant issue was the apparent lack of preparedness for a mine fire, with the state-government commissioned Hazelwood Mine Fire Inquiry noting that “all of the factors contributing to the ignition and spread of the fire were foreseeable. Yet it appears they were not foreseen” [2]. This failure to take appropriate measures to prevent a likely fire meant that the community likely saw the mine fire as having both natural and human causes. In line with the reviewers' suggestion, we have expanded the discussion at lines 404-412, including directions for future research.

***“Longer-term social outcomes after man-made or industrial disasters have the potential to be worse than in cases of natural disasters [85], however, it is at present unclear whether this also applies to academic outcomes. Furthermore, while there is little research available for assessing the relative impacts of different types of disasters on academic outcomes, it is reasonable to anticipate that more severe disasters will have a potentially greater impact on academic outcomes. For instance. if school infrastructure was destroyed, or if students were exposed to the trauma of having lost family or classmates in the event, then academic consequences might be expected to be worse than in the case of the Hazelwood mine fire. Accordingly, seeking to compare how different types of disaster shape academic outcomes would be a useful direction for future research.”***

Discussion: Line 377. Not sure if you are refereeing to a specific context. I identified at least 2 studies that would match this worldwide.

**Response**: We agree with the reviewer that we could be more specific in this assertion. We are not aware of any studies having used aggregated school level-data in this Bayesian multi-level meta-regression framework. A very simple illustrative case was used in Andrew Gelman’s book, Bayesian Data Analysis, which inspired this study. Please feel free to let us know if we missed anything.

“To the best of our knowledge, this is the first study using a Bayesian **meta-regression** method to evaluate **aggregated** school-level academic outcomes.”

Discussion: Line 382. I have read the response to a previous comment in this line, but I still think the analysis does not really evaluate the spatial profile of the impacts since the model does not incorporate a spatial term or a spatial structure specification.

**Response**: We agree that the model does not include a strictly defined spatial term. Our “spatial” term refers to geographical areas of low, medium and high exposure levels (see lines 419-420).

*“Firstly, it enables the evaluation of the spatial (****geographical regions of*** *no/low exposure to high exposure areas)”*

Although we can estimate the continuous spatial term (e.g., distance to the mine) and include a spatial and temporal interaction, it is not possible to interpret the results (there is both the direct interruption effect and the change of slope effect). We feel that it would be suitable for readers to consider that the method can model differential effects based on geographical location.

Limitations: The Limitations section needs to address specifically the potential risk of ecological bias. The impact can be significant (potential spurious associations due to analysis at the group rather than individual level) and the authors need to acknowledge this limitation. This also links to how the conclusions are presented. An ecological analysis cannot provide evidence of causality (rather a statistical evidence, or conclusions circumscribed to the context of the analysis).

**Response**: Thank you, we have now addressed this in the discussion. The interrupted time-series design aims at evaluating changes in population trends associated with an event, which is less likely to be confounded by individual-level factors except when the individual factor is associated with the event itself [3]. We have also included a control group to reduce the impact of time-varying confounders. In lines 422-426, we have added the benefits of including a control group in the analysis.

***“The inclusion of an appropriate control group of schools from areas with similar socioeconomic circumstances, but not exposed to smoke during the fire, countered the problem of potential time-varying confounders (e.g., changes in tests, education policy or other major interruptions) and enabled the impact of the mine fire on academic progress in the region to be clearly isolated [91]”***

In lines 474-475 we have included ecological bias as a potential limitation.

***“****Furthermore, the aggregated nature of the data precluded controlling for individual-level risk factors and,* ***hence, there is potential for ecological bias within the study.”***

**Reviewer #2:**

The project is based on an excellent idea: demonstrating how readily-available administrative data, when analysed in a sensible way, can provide insight into the effects of local disasters. As the authors state, an analysis of the Hazelwood mine fire could form a template for analyses of disasters elsewhere (where there is good admin data available). The basic design of the analysis, with two types of control groups plus before-after comparisons, is nice, in that it is easy to understand and has a good chance of isolating the actual effects of the disaster. The authors have done a good job of responding to comments from an earlier reviewer. In my opinion, however, a bit more work is required to bring the modelling itself, plus the description of the modelling, to the point where it would form a good template for analyses of other disasters. My reservations are as follows.

**Response**: Thank you for these positive comments. We have addressed the comments in relation to the paper below.

One of the fundamental motivations for using Bayesian hierarchical models is to stabilise estimates, and avoid coefficient estimates that merely reflect random variation. See, for instance, <https://www.tandfonline.com/doi/abs/10.1080/19345747.2011.618213>

**Response**: Fantastic suggestion. Gelman’s paper is a great reference. We have included additional discussion in lines 445-448.

***“However, fitting models with the Bayesian approach has additional advantages such as more stable and generalisable estimates, integration of existing knowledge using prior distributions, flexible compressions using posterior distributions and avoiding convergence and estimation problems of maximum likelihood methods [61,93].”***

The typical mechanism for achieving shrinkage is to put priors on variance terms that favour values near zero - see, for instance, <https://github.com/stan-dev/stan/wiki/Prior-Choice-Recommendations>**.** Some form of shrinkage arguably should be a default, and I would expect it to be included in a template for other analyses. However, judging by the description on pp11-12, the models in the paper do not take advantage of this possibility. Perhaps the authors could (1) explain why they did not build shrinkage into their analysis, or (2) amend the model?

**Response**: We believe the reviewer is referring to partial pooling of the random intercepts. Our two distributions for the random effects - one for the school random intercepts, another for the cohort random intercepts - each assume a single standard deviation term, on which we place a weakly informative prior. This common SD ensures some level of partial pooling across schools and cohorts. Our truncated normal prior for the random effects SD did use a mean of 10 rather than 0, which may be what the reviewer is referring to in their comment. The mean of 10 was chosen instead of 0 because there were slightly fewer high-performance schools and we did not want to underestimate this variation. However, the weekly prior distribution had very little impact on our results. See examples below:

Using the mean of 0 in prior distributions in the tutorial code.

Using the mean of 10 in prior distributions in the tutorial code.

We have included this in lines 238-240.

***“A mean of 0 is normally used for prior distributions of the random effects to take more advantage of the partial pooling effect of the multi-level model [61]. In our case, the mean of 10 was chosen as the high-performance schools were slightly underrepresented in the area and variations across all regional Victorian schools were used (based on a preliminary exploratory evaluation).”***

The reviewer has also reminded us to mention the possible usage of shrinkage priors, please see our updates in lines 448-451.

***“The choice of prior distributions did not impact our results, however, when there is high model complexity (e.g., higher numbers of potential confounding factors), penalising Bayesian models can be used with shrinkage prior distributions [94, 95].”***

ALGEBRAIC DESCRIPTION OF MODEL: I found the algebraic description of the model on pp10-11 difficult to follow. What is the 'e' subscript?

**Response**: The 'e' subscript represents exposure. These are six terms associated with exposure, which were listed separately to explain how to interpret mine fire exposure-related effects. To increase the readability of these notations, we have modified as follows:

- $\beta_{m,prior}$ and $\beta_{h,prior}$: prior mine fire differences for moderate and high exposure schools
- $\beta_{m,int}$ and $\beta_{h,int}$: mine-fire interruption effects
- $\beta_{m,trend}$ and $\beta_{h,trend}$: post-mine fire trend differences

See lines 214-230 for all the updates.

Given that the outcomes are time indexed, it seems like there should be a time subscript, but there isn't one. It seems like the time subscript may be included implicitly in beta and X, but this is confusing, and I'm not sure it's quite correct.

The model includes two temporal variables. One is the year (continuous variable) as a long-term trend, which is described as a confounder*.* Another temporal term is the $T_{post}$ - post-mine fire time variable (0 for 2008-2014, 1 for 2015, 2 for 2016 etc) which was included in the exposure time interaction term in the second equation, which is a standard interrupted time series parameterisation [4]. We have included the long-term trend term ($T$) in Equation (2) to make this clear.

I looked for the Stan code, to clarify what was going on, but the supplementary materials were not included with the submission - which may be a PLOS problem??

PLOS one places the Supplementarily Material at the end of the full-text (before the track-changed document). The data and Stan code can also be found on the data repository Zenodo: <http://doi.org/10.5281/zenodo.6613107>.

A more minor point - rather than beta X_{s,g}, where X is a matrix, you probably want x_{s,g} beta, where x_{s,g} is a row vector and beta is a column vector.

**Response**: Yes, it should be $\mathbf{X}_{\mathbf{s,g}}\beta$, this is corrected in Equation (1)

IMPORTANCE OF CONTROL GROUPS? The Abstract and Discussion emphasise the use of admin data, before-after comparisons, and Bayesian modelling. Arguably, the use of appropriate control groups is also an important part of the general template. The results would have been much less convincing if only values for the directly affected schools were shown. Perhaps this requires more emphasis? (And, if so, some thought about problems with using the design to study the effect of COVID on schooling.)

**Response**: We agree with this suggestion. In this particular case, schools in SA3 areas with similar socioeconomic profiles but not impacted by the mine-fire smoke were chosen for the control group. As suggested, the control group in the study is valuable for ensuring that the changes observed were not due to other time-varying factors such as changes in assessment content, funding policy, other major interruptions etc. The control group improves the robustness of the finding and we have included additional discussion in lines 422-426.

***“The inclusion of an appropriate control group of schools from areas with similar socioeconomic circumstances, but not exposed to smoke during the fire, countered the problem of potential time-varying confounders (e.g., changes in tests, education policy or other major interruptions) and enabled the impact of the mine fire on academic progress in the region to be clearly isolated [91].”***

MULTIPLE OUTCOMES: One important feature of the analysis is that it uses multiple outcomes. This creates some challenging problems of interpretation and inference in cases where different outcomes appear to be affected differently. There is not consensus on the best approach to take to multiple outcomes - see, for instance, <https://statmodeling.stat.columbia.edu/2022/05/18/doing-mister-p-with-multiple-outcomes/>. But maybe it deserves a bit more discussion, and the approach of the paper (separate models for each outcome) needs to be mentioned and defended?

**Response**: This is definitely worth additional discussion. From an educational psychology point of view, it would be remiss to combine all domain scores into a single score, as general developmental trends are different across domains. It would also be presumptuous to expect the impacts would be the same across all domains, as some domains might be more vulnerable to immediate changes and interruptions. These are the practical reasons behind modelling the outcomes separately. As the reviewer suggested there is currently no consensus on the best approach. We have included the following additional discussion in the limitation part and highlighted that when the assumption or interest is to understand a consistent impact, then the single multi-level model can be used, see lines 466-473.

***“Outcomes across multiple learning domains were compared which can introduce challenges for interpretation. In this study, it is likely that interruptions caused by the mine fire to school routines were distributed similarly across all learning domains; however, the level of impact of this disruption on students’ subsequent progression within each learning domain may differ. Therefore, each domain was evaluated separately to investigate for any differing impacts and trends between domains. Alternatively, if there was an assumption or interest in evaluating a consistent impact across learning domains, all could be included in one multilevel model with an additional nested level [61].”***

**References**

1.	Reifels L, Mills K, Dückers MLA, O'Donnell ML. Psychiatric epidemiology and disaster exposure in Australia. Epidemiology and Psychiatric Sciences. 2019;28(3):310-20. Epub 2017/09/27. doi: 10.1017/S2045796017000531.

2.	Teague B, Catford J, Petering S. Hazelwood Mine Fire Inquiry Report. Vol. 1. . Vic Government Printers, 2014. Available from: <https://www.parliament.vic.gov.au/file_uploads/8101_HAZ_Hazelwood_Mine_Inquiry_Report_BOOK_LR_f5Bp6wNh.pdf>

3.	Penfold RB, Zhang F. Use of interrupted time series analysis in evaluating health care quality improvements. Acad Pediatr. 2013;13(6 Suppl):S38-44. doi: <https://doi.org/10.1016/j.acap.2013.08.002>.

4.	Penfold RB, Zhang F. Use of Interrupted Time Series Analysis in Evaluating Health Care Quality Improvements. Academic Pediatrics. 2013;13(6, Supplement):S38-S44. doi: <https://doi.org/10.1016/j.acap.2013.08.002>.
