# Supplementary material for: Evaluating the impact of Hazelwood mine fire event on students’ educational development with Bayesian interrupted time-series hierarchical meta-regression
Source: PLoS One. 2023 Mar 1;18(3):e0281655. doi: 10.1371/journal.pone.0281655 (PMC9977026; doi:10.1371/journal.pone.0281655)
Supplement: Attachment — Submitted filename: Responses to Reviewers_V3.docx [file pone.0281655.s005.docx]

**Responses to Reviewers**

Our sincere thanks to the reviewers for providing such detailed and helpful comments on our manuscript. We have responded to each comment below and highlighted relevant changes in the revised manuscript.

**Reviewer #1:**

I appreciate the authors have considered my previous comments. From reviewing this current version of the manuscript, there are just two additional aspects that still need to be addressed before acceptance for publication:

**Response**: Thank you for these positive comments. We have addressed the comments in relation to the paper below.

In the response to the comparability to individual level analyses, I don’t agree that “the school-level distributions should obtain similar results to modelling outcomes based on individual students’ records”. The use of school level data implies an ecological model and I understand there is not a statistical approach to overcome this limitation. This was previously discussed in the first review. Reconsider to update the statement in line 183 to reflect accurately on this.

**Response**: Thanks for the suggestion. We may not make our argument clear enough here. We did not intend to infer that ecological models are the same as individual-level models (ecological bias is well-known and were discussed in the limitation part). Here we are refereeing to the fact that the meta-analysis, which considered both the means and SEs, is unbiased compared with including individual-level models without confounder adjustment. If only mean scores were used, then the results will be biased (smaller schools with wider distribution will be given more weights than there should be). We have modified the discussion to reflect this more clearly as follows:

*“This approach directly models distributions of students’ performance within schools using mean scores and standard errors,* ***which follows the meta-analysis approach*** *to ensure unbiased estimates that are comparable with the* ***individual-level model without confounder adjustment (assuming there is no ecological bias)****.”*

I think the discussion still needs more support on local studies on the lack of quality impact assessment in the Australian coal mining sector. Research on the impacts of coal mining in the country is scarce and it is of value to highlight previous relevant research. Consider again the references commented in the first review.

**Response**: We have included the recommended reference and discussion in the last review submission. We have modified this section according to the reviewer’s suggestion.

*“Fire incidents are common in coal mines [70] and, all too often, mining project impact assessments adopt a narrow range of health and wellbeing domains that omit consideration of* ***epidemiological evidence*** *and potential social consequences [71,* ***72****]. Accordingly, the impacts that events such as the Hazelwood mine fire can have on academic outcomes among school-aged young people warrant consideration within mine project planning and* ***impact assessment****, more generally, within disaster risk reduction planning and education department policies.”*

**Reviewer #2:**

I am satisfied that the authors seriously considered all the questions that I raised in my review. I do not always agree with the authors' choices. I continue to find the use of a truncated normal with mu=10 strange, in that it actually pulls the posterior away from zero in cases where the observed standard deviation is below 10. However, reasonable people can disagree on these things, and the authors note that the results are not sensitive to the choice of prior.

**Response**: Thank you for these positive comments. Our choice is perhaps more impacted by our prior beliefs when working with schools in the area. The schools in the region would likely be at least as diverse as if not more diverse than the rest of the regional areas. As the reviewer suggested It is more reassuring that prior distributions had very little impact on results. We have addressed the remaining comment below.

One minor suggestion: in the notation on 214-220, perhaps, in a Bayesian context, the terms 'prior' and 'post' are not ideal, as they invite confusion with prior and posterior distributions. Better to use 'before' and 'after'?

**Response**: We agree that the term “prior” can be easily confused with “prior distribution”. We have also noticed that we have used “pre” and “prior” interchangeably, which may also contribute to this confusion. It is slightly awkward to refer to the “post-mine fire trend” as “the trend after the mine fire”, so we decided to keep this and change “prior-” and “post-” mine fire to “pre-” and “post-” mine fire. Hopefully, this can avoid confusion as “pre and post-disaster” are commonly used terms.
